# The expression of miR‐125b in Nrf2‐silenced A549 cells exposed to hyperoxia and its relationship with apoptosis

**DOI:** 10.1111/jcmm.14808

**Published:** 2019-11-12

**Authors:** Xiaoyue Zhang, Xiaoyun Chu, Xiaohui Gong, Huilin Zhou, Cheng Cai

**Affiliations:** ^1^ Department of Neonatology Shanghai Children’s Hospital Shanghai Jiao Tong University Shanghai China

**Keywords:** bronchopulmonary dysplasia, miR‐125b, Nrf2, premature, siRNA

## Abstract

Bronchopulmonary dysplasia (BPD) is a chronic lung disease that affects the quality of life of infants. At present, premature exposure to hyperoxia for extended periods of time is believed to affect the development of lung tissue and vascularity, resulting in BPD. The oxidative stress caused by hyperoxia exposure is an important risk factor for BPD in premature infants. Nuclear factor E2‐related factor 2 (Nrf2) is an important regulator of antioxidant mechanisms. As a microRNA, microRNA‐125b (miR‐125b) plays an important role in cell proliferation, differentiation and apoptosis. Although the Nrf2/ARE pathway has been extensively studied, little is known about the regulatory role of microRNAs in Nrf2 expression. In this study, the expression levels of Nrf2 and miR‐125b in the lung tissues of premature Sprague Dawley (SD) rats and A549 cells exposed to hyperoxia were detected by quantitative real‐time polymerase chain reaction (qRT‐PCR), and the apoptosis of A549 cells was detected by flow cytometry. The results showed that Nrf2 and miRNA‐125b in the lung tissues of premature rats increased significantly upon exposure to hyperoxia and played a protective role. Nrf2 was suppressed by small interfering RNA (siRNA) in A549 cells, miR‐125b was similarly inhibited, and apoptosis was significantly increased. These results suggest that miR‐125b helps protect against BPD as a downstream target of Nrf2.

## 1 INTRODUCTION

Bronchopulmonary dysplasia (BPD) is a chronic lung disease (CLD) that seriously affects premature infants, especially very low birthweight infants (LBWI)/extremely low birthweight infants (ELBWI). At present, most scholars believe that premature infant exposure to hyperoxia and excessive production of reactive oxygen species (ROS) in vivo destroy the oxidation‐antioxidant balance, which is the main risk factor for BPD.^1^ In a recent study, newborn mice were exposed to hyperoxia, and stereological investigations were performed 14 days after birth. Hyperoxia differentially influenced the maturation of lung parenchyma. A significant retardation of morphological lung development leading to BPD‐like alterations, as indicated by different parameters, was observed.^2^ Nuclear factor E2‐related factor 2 (Nrf2) is a highly evolutionarily conserved transcription factor with a leucine zipper (bZIP), which contains the Cap'n'Collar (CNC) structure.^3^ When stimulated by oxidants, drugs or pathogens, Nrf2 and Kelch‐like ECH‐related protein 1 (Keap1) dissociate and bind to antioxidant reaction elements (AREs) in the nucleus to initiate the transcription of cytoprotective genes.^4^ Nrf2 activation in response to ROS‐induced lung injury in pre‐term concurs to the induction of certain number of antioxidant, anti‐inflammatory and detoxification pathway. These elicited protective effects are able to counteract/mitigate all multifaceted aspects of the disease and may support novel approaches for the management of BPD.^5^ miR‐125b belongs to the has‐miR‐125b family and consists of miR‐125b‐1 (located on chromosome 11 q24) and miR‐125b‐2 (located on chromosome 22 q21), which protect endothelial cells from apoptosis under oxidative stress.^6^ Lukiw and Pogue^7^ isolated microRNAs from HN cells exposed to magnesium sulphate (control), aluminium sulphate or aluminium plus iron sulphate. microRNA arrays showed that miR‐125b was up‐regulated by metal sulphate‐generated ROS, suggested that miR‐125b to be modulated by oxidative stress. However, the regulatory role of microRNAs (miRNAs) in Nrf2 expression is not fully understood. Recent studies have shown that miRNAs regulate Nrf2 directly or indirectly,^8^ which may be an important mechanism underlying miRNAs involvement in cell differentiation and apoptosis. miRNAs are involved in the regulation of intracellular redox and post‐transcriptional regulation of key components of the ROS/reactive nitrogen (RNS) pathway, including Nrf2.^9^ Jing Zhou et al^10^ showed that H_2_O_2_ activated the Nrf2 signalling pathway in a mouse corneal epithelial progenitor cell line (TKE2) and increased the expression of miR‐125b.

Nrf2/ARE is a key pathway in the antioxidant response in the human body. As important post‐transcriptional regulators, miR‐125b and Nrf2 play irreplaceable roles in proliferation, differentiation and regulation. We hypothesized that the expression of miR‐125b is significantly correlated with Nrf2 and has an effect on cell apoptosis. We elucidated the cellular defence mechanism of Nrf2 in the development of BPD and provided new ideas for the prevention and treatment of BPD in premature infants.

We hypothesized that (1) oxidative stress induced by hyperoxia exposure can lead to BPD in premature infants and trigger a series of antioxidant mechanisms, which involve miR‐125b as an important cell protective factor and that (2) miR‐125b has an important role in the antioxidant and anti‐apoptotic effects of the Nrf2 pathway.

## 2 MATERIALS AND METHODS

### 2.1 Ethics approval

The use and care of laboratory rodents was performed according to the Animal Laboratory Center of Pediatrics, Children's Hospital of Fudan University and approved by the Committee of Animal Laboratory Management and Ethics, Shanghai Children's Hospital.

### 2.2 Animal experimental grouping

In total, 250‐300 g healthy adult SPF Sprague Dawley (SD) rats, 25 females and 5 males, were raised in cages. The animal licence number SCXK (Shanghai) 2018‐0006 was provided by Shanghai Xipuer‐Bikai Laboratory Animal Co. Ltd. Eighty premature SD rats with a gestational age (GA) of 21 days (full‐term GA 22 days) were randomly divided into two groups: the air group and hyperoxia group. The premature rats in the air group were fed in a normal pressure room (21% O_2_), and the premature rats in the hyperoxia group were fed in a 90 × 60 × 45 cm homemade plexiglass oxygen chamber. The oxygen flow rate in the oxygen chamber was 1‐3 L/min. The oxygen concentration curve in the chamber was recorded by a medical digital oxygen monitor within 24 hours, and the oxygen volume fraction in the chamber was kept at 80 ± 5% O_2_. Female rats that gave birth naturally were used as surrogate suckling mice. At 1, 4, 7, 10 and 14 days after exposure, the lung tissues of premature rats were placed in 5‐mL enzyme‐free cryopreservation tube and cooled rapidly with liquid nitrogen. The specimens were transferred to a –80°C freezer for subsequent PCR experiments.

### 2.3 Transfection and screening of A549 cells

On the day before transfection, 5 × 10^4^ cells were inoculated in a 24‐well plate, and 10% FBS and Dulbecco's modified Eagle's medium (DMEM) with antibiotics were added. After 24 hours, cell confluence up to 40%‐70% was achieved. Next, 50, 100 and 200 nmol/L siRNA was added to the DMEM serum‐free medium. Then, 1 µL Lipofectamine 2000 reagent diluted with 50 µL serum‐free DMEM was added and incubated at room temperature for 5 minutes, and diluted siRNA and Lipofectamine reagents were mixed and incubated for 20 mintes to form the siRNA/Lipofectamine (or DNA/Lipofectamine) complex, and the complex was added to the culture plate containing the cells and medium and transfected for 8‐12 hours.

The screening experiment was divided into three groups: siRNA‐1, siRNA‐2 and siRNA‐3. The blank control group had no siRNA at the time of transfection. Lipofectamine 2000 transfection reagent was used according to the kit instructions. After transfection of three different siRNA Nrf2 plasmids into A549 cells, changes in Nrf2 expression levels were detected by qRT‐PCR. The siRNA that most efficiently suppressed Nrf2 was selected for follow‐up experiments.

A549 cells were randomly divided into four groups: non‐interference air group, non‐interference hyperoxia group, air group after interference and hyperoxia group after interference. The hyperoxia group was exposed to a highly pure gas mixture of 3 L/min 92% O_2_ and 5% CO_2_ and sealed after 10 minutes. Both groups were placed in an incubator (37°C, 5% CO_2_). Forty‐eight hours after incubation, total RNA was extracted from A549 cells.

### 2.4 Expression of Nrf2, miR‐125b and IL‐1β detected by qRT‐PCR

Nrf2, miR‐125b and IL‐1β were detected by qRT‐PCR. The control genes were β‐Actin and glyceraldehyde‐3‐phosphate dehydrogenase (GAPDH). The total RNA of A549 was extracted by the TRIzol method. qRT‐PCR was carried out using SYBR Green technology. The samples were pre‐amplified at 95°C for 10 minutes, after which they were amplified for 40 cycles at 95°C for 2 seconds, 60°C for 20 seconds and 70°C for 10 seconds. qRT‐PCR was performed on a PCR instrument (Bio‐Rad CFX96). The relative expression of target gene mRNA in the samples was determined by 2^−△△Ct^.

### 2.5 Western blotting analysis

Western blotting analysis was performed to assess protein abundance as described in our previous study. The primary antibody was Nrf2, and the secondary antibody was horseradish peroxidase (HRP) goat‐anti‐rabbit. Bio‐Rad Image Lab Software (Version 5) was used for the analysis. After transfection of the Nrf2 siRNA plasmid into A549 lung cancer cells, an appropriate amount of cell lysate was added, and proteins from the nucleus and cytoplasm were collected and extracted. The collected proteins were mixed with buffer solution and then cooled on ice after 5 minutes in a water bath at 100°C. A separation gel and concentration gel were prepared for electrophoresis for 90 minutes. Next, the polyvinylidene fluoride (PVDF) membrane was removed and placed in a culture dish, and an appropriated amount of buffer [PBS buffer containing 5% (w/v) skim milk powder] was added and incubated at room temperature for 1‐2 hours. The culture dish was replaced, 10 mL of the abovementioned buffer solution was added, and the primary antibody diluted at 1:1000 was added. Then, the membranes were incubated overnight at 4°C, washed three times, transferred to another culture dish and incubated with the secondary antibody buffer containing 5% skim milk powder and with the secondary antibody (HRP goat‐anti‐rabbit IgG) at 1:5000. The membranes were incubated at room temperature for 1 hour. Then, the PVDF membrane was transferred to another culture dish and washed three times with an appropriate amount of secondary antibody buffer. Finally, the Electrochemiluminescence (ECL) substrate developer (Thermo) and the substrate developer were added for imaging.

### 2.6 Flow cytometry

Flow cytometry was used to detect the effect of hyperoxia and siRNA targeting Nrf2 on apoptosis of A549 cells. Cells in the logarithmic growth phase were digested by trypsin and then made into a cell suspension. The cells were cultured at 37°C for 48 hours, the culture medium was removed, and the cells were washed twice with PBS. After trypsin digestion of the adherent cells, the cells were collected. The cells were washed twice with pre‐cooled PBS at 4°C and then centrifuged for 10 min (1000 r/min, 40°C). Then, the supernatant was discarded, the cells were collected, and the procedure was repeated for twice. The cell suspension was suspended again in 200 μL binding buffer, and 10 μL Annexin V‐FITC was added to the cell suspension to mix evenly for 15 minutes at room temperature in the dark. Then, 300 μL binding buffer was added to the cell suspension (total reaction volume 500 μL) for 15 minutes at room temperature; flow cytometry was used for detection.

### 2.7 Statistics

The data were analysed by SPSS 20.0 statistical software, and the results represent the mean ± SD of independent experiments. Relative expression levels among the siRNA interference group (including siRNA‐1, siRNA‐2 and siRNA‐3), the blank group, air group and hyperoxia group were tested by Student's t test. One‐way ANOVA was used between the two groups on apoptosis of A549 cells. The results with *P < *.05 were considered significant.

## 3 RESULTS

### 3.1 Expression of Nrf2 in the lung tissues of premature SD rats

**Figure 1:**
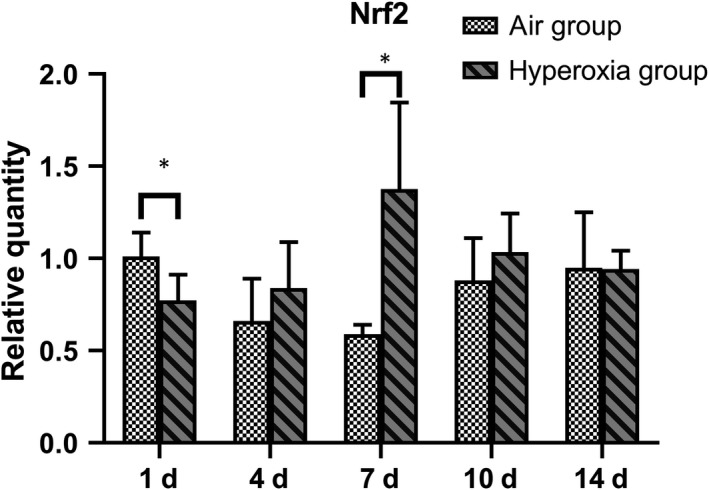
Changes in Nrf2 expression in lung tissue of premature rats at different time‐points. qRT‐PCT assays of lung Nrf2 levels in premature rats exposed to air or hyperoxia. Compared with the air group, Nrf2 increased gradually after birth and reached its peak on the 7th day and then decreased. There were significant differences on the 7th day. Data represent the mean ± SD *P < .05

Compared with that of the air group, the expression of Nrf2 in the hyperoxia group was significantly increased on the 7th day (*P* < .05), but no significant differences were observed on the 4th, 10th and 14th day (*P* > .05) (Table 1 and Figure 1).

**Table 1:** Changes in Nrf2 mRNA expression in lung tissues of premature rats at different time‐points in two groups

Groups	n	1 d	4 d	7 d	10 d	14 d
Air group	8	1.006 ± 0.128	0.664 ± 0.234	0.596 ± 0.047	0.879 ± 0.228	0.953 ± 0.305
Hyperoxia	8	0.774 ± 0.139	0.844 ± 0.245	1.374 ± 0.474	1.034 ± 0.206	0.942 ± 0.104
*t*		2.979	−1.096	−3.650	−1.209	0.087
*P*		.014	.305	.021	.255	.933

### 3.2 Expression of miR‐125b in the lung tissues of premature SD rats

**Figure 2:**
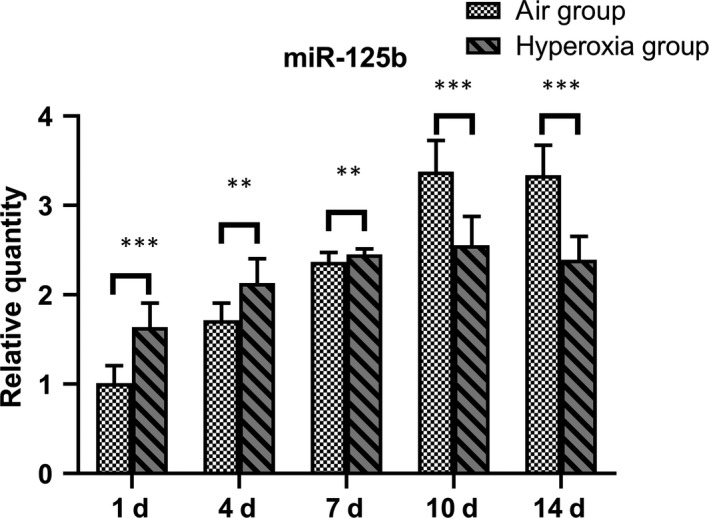
Changes in expression of microRNA‐125b in lung tissue of premature rats at different time‐points. Both air group and hyperoxia group, miR‐125b increased gradually after birth. Compared with the air group, there were statistical differences at each time‐point. miR‐125b reached its peak on 10th, which is similar to Nrf2. Data represent the mean ± SD **P < 0.01, ***P ＜ .001

Compared with that of the air group, the miR‐125b increased significantly on the 1st, 4th and 7th days, and decreased significantly on the 10th and 14th days (*P* < .05) (Table 2 and Figure 2).

**Table 2:** Changes in the expression of miR‐125b in lung tissue of premature rats at different time‐points in two groups

Groups	n	1 d	4 d	7 d	10 d	14 d
Air group	8	1.010 ± 0.200	1.716 ± 0.194	2.369 ± 0.105	3.380 ± 0.349	3.340 ± 0.336
Hyperoxia	8	1.638 ± 0.217	2.131 ± 0.275	2.450 ± 0.064	2.554 ± 0.324	2.329 ± 0.263
*t*		−6.033	−3.484	−2.970	4.912	6.701
*P*		＜.001	.004	.001	＜.001	＜.001

### 3.3 Nrf2 suppression by siRNA

**Figure 3:**
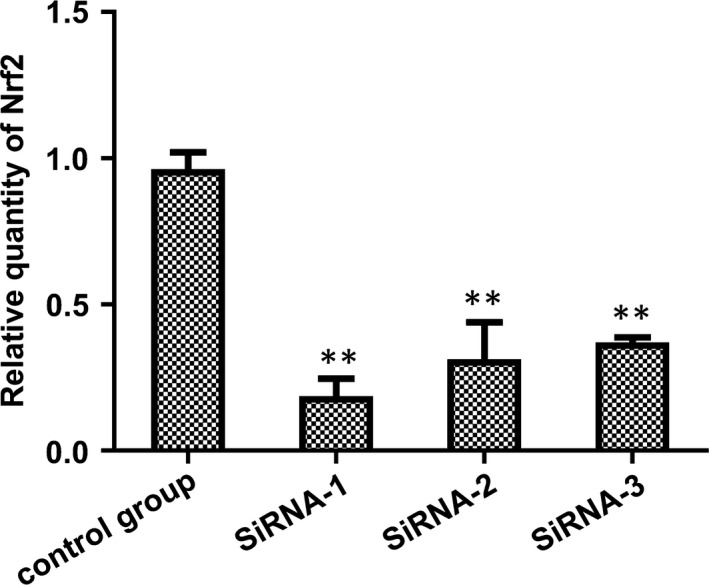
Nrf2 suppressed by siRNA. siRNA designed by GenePharma. The control group was that Nrf2 not suppressed by siRNA. siRNA1, siRNA2 and siRNA3 represent three different siRNAs transfected into A549 cells, respectively. Their primer sequences are shown in Table 4. Data represent the mean ± SD **P < .01

**Figure 4:**
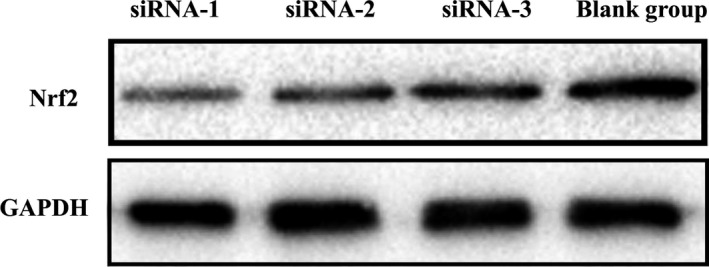
Nrf2 suppressed by siRNA for 48 h and subsequently subjected to protein isolation. Nrf2 and GAPDH (as reference gene) were measured by Western blot analysis. Molecular weight of Nrf2 is 70 kD including tags and GAPDH is 146 kD

siRNA‐1 had the highest inhibitory efficiency (80.58 ± 3.51%) (Tables 3 and 4 and Figure 3). The protein levels of the control GAPDH and Nrf2 are shown below (Figure 4). The result shows that siRNA plasmid transfection cells for 48 hours, the inhibition efficiency was the highest, so we chose hyperoxia exposure for 48 hours as the time‐point for subsequent experiments.

**Table 3:** Detection and screening of the most efficient target for siRNA silencing Nrf2 by qRT‐PCR

siRNA‐1 (n = 3)	siRNA‐2 (n = 3)	siRNA‐3 (n = 3)	Blank group (n = 3)
0.187 ± 0.059	0.314 ± 0.126	0.370 ± 0.018	0.963 ± 0.057

**Table 4:** Primers of siRNA‐1, siRNA‐2 and siRNA‐3

Gene name	Primer	Sequence(5’‐3’)
siRNA‐1	Sense	5’‐AUACUUCUCGACUUACUCCAA‐3’
	Antisense	5’‐ GGAGUAAGUCGAGAAGUAUUU‐3’
siRNA‐2	Sense	5’‐ AAACGUAGCCGAAGAAACCUC‐3’
	Antisense	5’‐ GGUUUCUUCGGCUACGUUUCA‐3’
siRNA‐3	Sense	5’‐ AAUAUUAAGACACUGUAACUC‐3’
	Antisense	5’‐ GUUACAGUGUCUUAAUAUUGA‐3’

### 3.4 Expression of Nrf2 and IL‐1β in A549 cells

**Figure 5:**
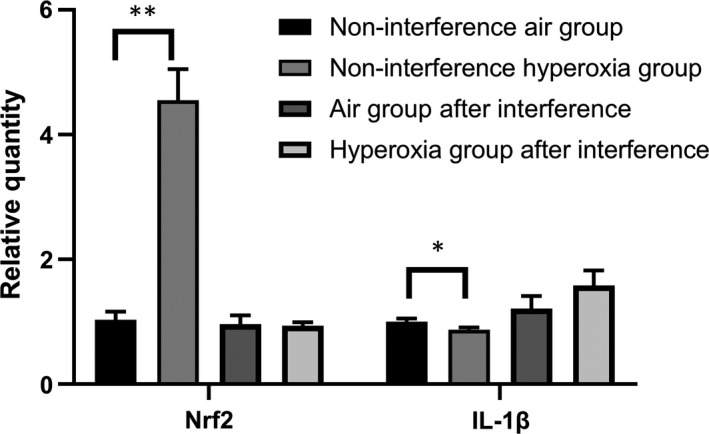
Nrf2 and IL‐1β in A549 while Nrf2 suppressed by siRNA and hyperoxia exposed. While A549 exposed to hyperoxia, Nrf2 increased significantly but IL‐1β decreased. After transfection of siRNA into A549 cells, the expression of Nrf2 was decreased. When Nrf2 was suppressed, then A549 cells were exposed to high concentration of oxygen, and there was no significant difference in the expression of Nrf2, but the expression of IL‐1β was increased. Data represent the mean ± SD *P ＜ .05, **P ＜ .01, ***P ＜ .001

The expression of IL‐1β in the non‐interference hyperoxia group after hyperoxia exposure at 48 hours was significantly different (*t* = 12.96, *P* = .028), and Nrf2 increased at the same time. When Nrf2 was suppressed by siRNA, the expression of IL‐1β increased and shows no significant difference after hyperoxia exposure (*t* = −2.031, *P* = .114) (Figure 5).

### 3.5 Influences of hyperoxia and siRNA on the expression of Nrf2 and miR‐125b in A549 cells

**Figure 6:**
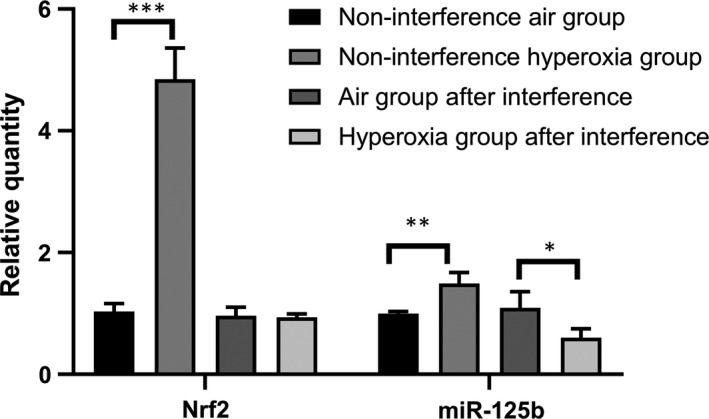
Nrf2 and miR‐125b increased significantly in A549 cells exposed to hyperoxia, and miR‐125b decreased while Nrf2 suppressed by siRNA after A549 cells exposed to hyperoxia, suggested that miR‐125b may be regulated by Nrf2, and participates in protection mechanism. Data represent the mean ± SD *P ＜ .05, **P ＜ .01, ***P ＜ .001

Compared with that of the non‐interference air group, the expression of Nrf2 and miR‐125b in the non‐interference hyperoxia group increased significantly (*P < *.05). Compared with that of the air group after interference, the expression of Nrf2 showed no significant change in the hyperoxia group (*t* = 0.070, *P* = .804) after 48 hours of hyperoxia exposure, and the expression of miR‐125b decreased significantly (*t* = 7.891, *P* < .05) (Table 5 and Figure 6).

**Table 5:** Influence of hyperoxia and siRNA silencing on the expression of Nrf2 and miR‐125b in A549 cells

Group (n = 3)	Nrf2	miR‐125b
Non‐interference air group	1.023 ± 0.129	0.996 ± 0.039
Non‐interference hyperoxia group	4.849 ± 0.515	1.491 ± 0.182
*t*	−12.616	−5.164
*P*	＜.001	.007
Air group after interference	0.961 ± 0.142	1.093 ± 0.265
Hyperoxia group after interference	0.937 ± 0.057	0.601 ± 0.147
*t*	0.070	7.891
*P*	.804	.048

### 3.6 The influence of siRNA targeting Nrf2 and hyperoxia on A549 cell apoptosis

**Figure 7:**
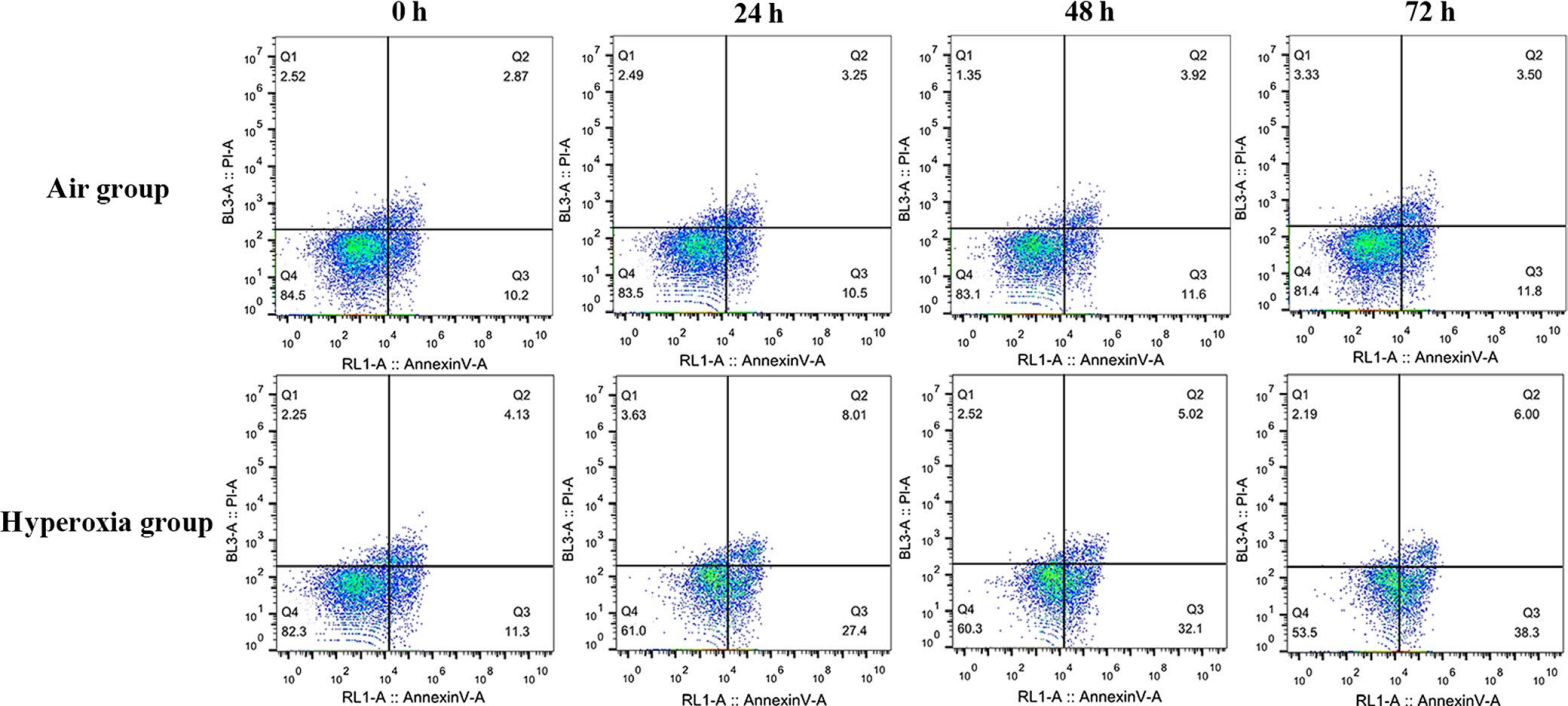
Apoptosis of A549 cells exposed to hyperoxia for 0, 24, 48 and 72 h after silencing Nrf2. Compared with the air group, the apoptotic fraction of A549 cells in the hyperoxia group after Nrf2 silencing was significantly higher at 24, 48 and 72 h (*F* = 886.878, 1205.854, 4470.786, *P* < .05), and these are 27.650 + 0.254% vs 11.000 ± 0.707%, 31.950 ± 0.212 vs 12.150 ± 0.778% and 38.450 ± 0.212% vs 12.150 ± 0.495%, respectively. One‐way ANOVA was used between the two groups

After Nrf2 was suppressed by siRNA, the apoptotic percentage of A549 cells in the hyperoxia group increased significantly at each time‐point (*P* < .05) compared with air group. The values were *F* = 886.878, 1205.854 and 4470.786 (*P* < .05), and these are 27.650 + 0.254% vs 11.000 ± 0.707%, 31.950 ± 0.212 vs 12.150 ± 0.778% and 38.450 ± 0.212% vs 12.150 ± 0.495%, respectively (Figure 7).

## 4 DISCUSSION

At present, the pathogenesis of BPD is still unclear. Infection and inflammation are believed to play an important role in BPD. New diagnostic criteria for BPD have been developed by the National Institute of Child Health and Human Development (NICHD); the main pathological features are alveolar and pulmonary microvascular dysplasia, which is characterized by alveolar reduction, enlargement, simplification of the alveolar structure and abnormal morphology of pulmonary microvessels, but alveolar and respiratory tract injury and fibrosis are mild.^11^ In recent years, many studies have proven that hyperoxia exposure is an important factor affecting the development of airway and pulmonary vascular anomalies.^12^ At present, disruption of oxidation/antioxidant mechanisms in the body is believed to interfere with alveolarization and pulmonary vascular development during early lung development, which is one of the pathogenic mechanisms of BPD.^13^ In the immune system, there is a dynamic balance between pro‐inflammatory cytokines and anti‐inflammatory cytokines. Long‐term exposure to high concentrations of oxygen activates and releases many inflammatory cytokines, resulting in endothelial peroxidation, increased vascular permeability and aggravated alveolar interstitial and airway oedema.^14^ Free radicals such as ROS and RNS produced by oxidative stress directly or indirectly attack cellular DNA, leading to cell degeneration and apoptosis.^15^ Therefore, enhancing the antioxidation ability and reducing ROS production are key to reducing the incidence of BPD and are important for improving the survival rate and quality of life of premature infants.^16^

The periods of lung development include the embryonic, pseudoglandular, canalicular, saccular and alveolar periods. At present, the animal models of BPD are mainly generated by hyperoxia exposure, and rodents are the most widely used model. For example, the pregnancy cycles of SD rats are relatively shorter than those of other models, and rats commence alveolarization ex utero and are born at a similar stage of lung development to that of a very pre‐term human infant (saccular stage).^17^ Previous studies have shown that 95% O_2_ exposure resulted in a pulmonary inflammatory response and fibrosis in neonatal rats.^18^ Alveolar epithelial cells play an important role in maintaining lung integrity and homeostasis in vivo. Alveolar epithelial type II cells (AECII) show potential epithelial repair and regeneration. Primary human AECII cells are difficult to isolate and culture, while A549 cells have similar biological characteristics to AECII cells, and primary cultured AECII cells are mutable and unsuitable for transfection. As mentioned above, since A549 cells were used to replace ACEII cells in vitro, the results do not represent the risk of the general population. Therefore, in this study, we established an animal model of BPD through neonatal rats exposed to hyperoxia and suppressed Nrf2 by siRNA in A549 cells instead of AECII cells. We explored the role of Nrf2 in BPD in animal and cell models and provided new ideas for the prevention and treatment of BPD.

Nrf2 is located on chromosome 2q31.2 and belongs to the CNC subfamily containing bZIP transcription factors. This group contains nuclear factor erythroid‐derived 2 (NFE2) and the NFE2‐related factors Nrf1, Nrf2 and Nrf3.^19^ Nrf2 is regulated by Kelch‐like ECH‐related protein 1 (Keap1), which controls the expression of key components of the glutathione (GSH) and thioredoxin (TXN) antioxidant system, as well as enzymes involved in NADPH regeneration, ROS and xenobiotic detoxification, and heme metabolism, thus playing a fundamental role in maintaining the redox homeostasis of the cell.^20^ As shown by a complementary DNA (cDNA) microarray in lung tissues, Nrf2‐mediated protection against hyperoxia‐induced acute lung injury (ALI) may be mediated by transcriptional regulation of genes related to DNA replication, the cell cycle, various metabolic pathways and small molecules, cell‐to‐cell interactions and redox homeostasis in neonatal mouse lung tissues during the cystic phase. *Nrf2* deficiency in immature lung aggravated lung injury and alveolar arrest induced by hyperoxia in neonates.^21^ Studies have shown that Nrf2 increased survival in hyperoxic conditions and attenuated hyperoxia‐induced alveolar growth inhibition in newborn mice.^22^ Therefore, as an antioxidant factor, Nrf2 has an important protective effect on oxidative stress induced by hyperoxia exposure in the respiratory tract.

miRNAs are non‐coding single‐stranded RNA molecules that post‐transcriptionally regulate target genes by binding specifically to the 3' untranslated region of target gene mRNAs. Prolonged hyperoxia alters the expression of miRNAs during normal lung development. These data support the hypothesis that the dynamic regulation of miRNAs plays a prominent role in the pathophysiology of BPD.^23^ The miR‐125 family is involved in many cellular processes, including cell differentiation, proliferation, metastasis, apoptosis and immune defence, and is composed of three homologs: hsa‐miR‐125a, hsa‐miR‐125b‐1 and hsa‐miR‐125‐2.^24^ Compared with miR‐125a, miR‐125b has been more extensively studied, and several targets of miR‐125b regulating cell survival, proliferation and differentiation have been suggested and experimentally confirmed.^25^ Up‐regulation of miR‐125b expression maintained the body weight and survival of ALI mice and significantly reduced LPS‐induced pulmonary inflammation.^26^ Another study showed that increased miR‐125b through toll‐like receptor 4(TLR4) affected mitochondrial respiration and dynamics through BIK and MTP18 silencing, respectively, promoting pro‐inflammatory activation and apoptosis of monocytes.^27^ Nrf2 has attracted increased attention as a protective transcription factor. Chromatin immunoprecipitation (ChIP)‐sequencing experiments were conducted in lymphoid cells treated with a dietary isothiocyanate and sulforaphane (SFN), and Brian N et al found that several miRNAs contain NRF2‐bound genomic regions and that 96% of these regions contain NRF2‐regulatory sequence motifs, showing that they can be regulated by Nrf2.^28^

In this study, the expression of Nrf2 in premature neonatal rats in the hyperoxia group was higher than that in the air group on days 1 and 7 (*P* < .05) and increased significantly on day 7, but no significant differences were observed on days 4, 10 and 14 (*P* > .05). Compared with that of the air group, the expression of miR‐125b increased significantly at days 1, 4 and 7 but decreased significantly on days 10 and 14 (*P* < .05). These results indicated that Nrf2 participates in the antioxidant response when stimulated by high concentrations of oxygen as an important antioxidant regulator. The expression of Nrf2 and miR‐125b in hyperoxia group increased gradually at first and then decreased. In acute myeloid leukaemia, a study showed that by decreasing the expression of miR‐125B and increasing the expression of miR‐29B, the cells showed increased sensitivity to apoptotic stimuli.^29^ miR‐125b‐5p regulates GSH levels and hence acetaminophen‐induced ALF by modulation of the Keap1‐Nrf2 signalling pathway. However, Nrf2 may also contribute to further changes in miR‐125b levels via a feedback loop as reported previously.^30^ Interleukin‐1 (IL‐1) represents a group of 17‐20 kD cytokines with a broad range of biological functions centred on the generation and maintenance of inflammatory processes. The best‐known members are IL‐1α, IL‐1β and IL‐1Ra (receptor antagonist).^31^ The ultimate mechanism of BPD development is pulmonary inflammation, which may be triggered by insults such as mechanical ventilation, antenatal and post‐natal infections and hyperoxia. The concentration of IL‐1β in the initial tracheal aspirate obtained from the lungs of pre‐term infants within the first hour of life could predict an adverse clinical course and short‐term outcome.^32^ In our study, the expression of IL‐1β in the non‐interference hyperoxia group after hyperoxia exposure at 48 hours was significantly different (*t* = 12.96, *P* = .028), and Nrf2 increased at the same time. After Nrf2 silencing by siRNA, the expression of IL‐1β increased and show no significant difference after hyperoxia exposure (*t* = −2.031, *P* = .114); therefore, Nrf2 can significantly inhibit the expression of IL‐1β, regulate the inflammatory response and play a role in cell protection.

Therefore, we conducted further studies. The apoptotic fraction increased with the prolongation of hyperoxia exposure when Nrf2 was suppressed by siRNA, showing that Nrf2 participates in the cell antioxidant response and prevents further damage to the body when stimulated by oxidative stress as a self‐protective mechanism. Compared with that of the non‐interference air group, the expression of Nrf2 and miR‐125b in the non‐interference hyperoxia group increased significantly after hyperoxia exposure at 48 hours. Compared with that of the air group after interference, the expression of Nrf2 did not change significantly, but the expression of miR‐125b decreased significantly (*t* = 7.891, *P* < .05) in the hyperoxia group after interference. This difference indicates that Nrf2 and miR‐125b increased in the cells after hyperoxia exposure, and miR‐125b decreased significantly when Nrf2 was suppressed by siRNA, showing that miR‐125b may be the downstream target of Nrf2. Nrf2‐ARE binding regulates the expression of more than 200 genes involved in cellular antioxidant and anti‐inflammatory defence.^33^ In the in silico* *promoter analysis using rVISTA, a conserved putative ARE sequence was identified in the promoter regions of *miR‐125b1* or* miR‐125b2;* consistently, treatment of NRK52E cells with sulforaphane (SFN, another activator of Nrf2) increased miR‐125b, which indicates that Nrf2 regulates miR‐125b through AREs.^34^

## 5 CONCLUSION

Both Nrf2 and miR‐125b are important factors in the antioxidant response. The increased expression of Nrf2 and miR‐125b in premature rats and A549 cells induced by hyperoxia exposure is involved in mitigating oxidative stress‐induced cell damage. miR‐125b plays a role in cellular protective mechanisms as a downstream target of Nrf2, but the specific mechanism of Nrf2 regulating miR‐125b has not yet been fully elucidated. Strategies to rationally regulate the expression of Nrf2 and miR‐125b to prevent and treat BPD require further study.

## CONFLICT OF INTEREST

The authors declare that they have no conflict of interest.

## AUTHORS’ CONTRIBUTIONS

ZXY, CC and GXH: made substantial contributions to the conception and design, acquisition of data or analysis and interpretation of data. ZXY, CXY, ZHL and CC: were involved in drafting the manuscript or revising it critically for important intellectual content. GXH and CC: revised the manuscript and gave the final approval of the version to be published. The authors agree to be accountable for all aspects of the work in ensuring that questions related to the accuracy or integrity of any part of the work are appropriately investigated and resolved. All authors read and approved the final manuscript.

## Data Availability

The data sets used and/or analysed during the current study are available from the corresponding author on reasonable request.

## References

[1] Wang J , Dong W . Oxidative stress and bronchopulmonary dysplasia. Gene. 2018;678:177‐183. 30098433 10.1016/j.gene.2018.08.031

[2] Schmiedl A , Roolfs T , Tutdibi E , et al. Influence of prenatal hypoxia and postnatal hyperoxia on morphologic lung maturation in mice. PLoS ONE ONE. 2017;12(4):e0175804. 10.1371/journal.pone.0175804 PMC5398543 28426693

[3] Loboda A , Damulewicz M , Pyza E , et al. Role of Nrf2/HO‐1 system in development, oxidative stress response and diseases: an evolutionarily conserved mechanism. Cell Mol Life Sci. 2016;73(17):3221‐3247. 27100828 10.1007/s00018-016-2223-0 PMC4967105

[4] Zhao H , Eguchi S , Alam A , et al. The role of nuclear factor‐erythroid 2 related factor 2 (Nrf‐2) in the protection against lung injury. Am J Physiol Lung Cell Mol Physiol. 2017;312(2):L155‐L162. 27864288 10.1152/ajplung.00449.2016

[5] Amata E , Pittala V , Marrazzo A , et al. Role of the Nrf2/HO‐1 axis in bronchopulmonary dysplasia and hyperoxic lung injuries. Clin Sci. 2017;131(14):1701‐1712. 28667068 10.1042/CS20170157

[6] Wei M , Gan L , Liu Z , et al. MiR125b‐5p protects endothelial cells from apoptosis under oxidative stress. Biomed Pharmacother. 2017;95:453‐460. 28865365 10.1016/j.biopha.2017.08.072

[7] Lukiw WJ , Pogue AI . Induction of specific micro RNA(miRNA) species by ROS‐generating metal sulfates in primary human brain cells. J Biol Chem. 2007;101(9):1265‐1269. 10.1016/j.jinorgbio.2007.06.004 PMC2080079 17629564

[8] Kurinna S , Werner S . NRF2 and microRNAs: new but awaited relations. Biochem Soc Trans. 2015;43(4):595‐601. 26551699 10.1042/BST20140317

[9] Paladino S , Conte A , Gaggiano R , et al. Nrf2 pathway in age‐related neurological disorders: insights into MicroRNAs. Cell Physiol Biochem. 2018;47(5):1951‐1976. 29969760 10.1159/000491465

[10] Zhou J , Ge L , Jia C , et al. ROS‐mediated Different homeostasis of murine corneal epithelial progenitor cell line under oxidative stress. Sci Rep. 2016;6(1):2. 27805062 10.1038/srep36481 PMC5090348

[11] Altit G , Dancea A , Renaud C , et al. Pathophysiology, screening and diagnosis of pulmonary hypertension in infants with bronchopulmonary dysplasia‐ A review of the literature. Paediatr Respir Rev. 2017;23:16‐26. 27986502 10.1016/j.prrv.2016.11.002

[12] Pabelick CM , Thompson MA , Britt RJ . Effects of hyperoxia on the developing airway and pulmonary vasculature. Adv Exp Med Biol. 2017;967:179‐194. 29047087 10.1007/978-3-319-63245-2_11

[13] Silva DM , Nardiello C , Pozarska A , et al. Recent advances in the mechanisms of lung alveolarization and the pathogenesis of bronchopulmonary dysplasia. Am J Physiol Lung Cell Mol Physiol. 2015;309(11):L1239‐L1272. 26361876 10.1152/ajplung.00268.2015

[14] Perrone S , Tataranno ML , Buonocore G . Oxidative stress and bronchopulmonary dysplasia. J Clin Neonatol. 2012;1(3):109‐114. 24027702 10.4103/2249-4847.101683 PMC3762019

[15] Kumar H , Lim HW , More SV , et al. The role of free radicals in the aging brain and Parkinson's Disease: convergence and parallelism. Int J Mol Sci. 2012;13(8):10478‐10504. 22949875 10.3390/ijms130810478 PMC3431873

[16] Huang B , Li Q , Xu S , et al. Substance P protects against hyperoxic‐induced lung injury in neonatal rats. Exp Lung Res. 2015;41(1):12‐20. 25275819 10.3109/01902148.2014.959140

[17] Ambalavanan N , Morty RE . Searching for better animal models of BPD: a perspective. Am J Physiol Lung Cell Mol Physiol. 2016;311(5):L924‐L927. 27663992 10.1152/ajplung.00355.2016 PMC5130540

[18] Zeng X , Zhou X , Yue S . Methods for establishing animal model of bronchopulmonary dysplasia and their evaluation. Chinese J Contemp Pediatr. 2017;19(1):121‐125. 10.7499/j.issn.1008-8830.2017.01.020 PMC7390119 28100335

[19] Sykiotis GP , Bohmann D . Stress‐activated cap'n'collar transcription factors in aging and human disease. Sci Signal. 2010;3(112):re3. 20215646 10.1126/scisignal.3112re3 PMC2991085

[20] Tonelli C , Chio I , Tuveson DA . Transcriptional regulation by Nrf2. Antioxid Redox Signal. 2018;29(17):1727‐1745. 28899199 10.1089/ars.2017.7342 PMC6208165

[21] Cho HY , Wang X , Li J , et al. Potential therapeutic targets in Nrf2‐dependent protection against neonatal respiratory distress disease predicted by cDNA microarray analysis and bioinformatics tools. Curr Opin Toxicol. 2016;1:125‐133. 28920101 10.1016/j.cotox.2016.10.006 PMC5596881

[22] McGrath‐Morrow S , Lauer I , Yee M , et al. Nrf2 increases survival and attenuates alveolar growth inhibition in neonatal mice exposed to hyperoxia. Am J Physiol Lung Cell Mol Physiol. 2009;296(4):L565‐L573. 19151108 10.1152/ajplung.90487.2008 PMC2670765

[23] Dong J , Carey WA , Abel S , et al. MicroRNA‐mRNA interactions in a murine model of hyperoxia‐induced bronchopulmonary dysplasia. BMC Genom. 2012;13:204. 10.1186/1471-2164-13-204 PMC3410783 22646479

[24] Sun YM , Lin YK , Chen YQ . Diverse functions of miR‐125 family in different cell contexts. J Hematol Oncol. 2013;6:6. 23321005 10.1186/1756-8722-6-6 PMC3566921

[25] Shaham L , Binder V , Gefen N , et al. MiR‐125 in normal and malignant hematopoiesis. Leukemia. 2012;26(9):2011‐2018. 22456625 10.1038/leu.2012.90

[26] Guo Z , Gu Y , Wang C , et al. Enforced expression of miR‐125b attenuates LPS‐induced acute lung injury. Immunol Lett. 2014;162(1):18‐26. 25004393 10.1016/j.imlet.2014.06.008

[27] Duroux‐Richard I , Roubert C , Ammari M , et al. miR‐125b controls monocyte adaptation to inflammation through mitochondrial metabolism and dynamics. Blood. 2016;128(26):3125‐3136. 27702798 10.1182/blood-2016-02-697003 PMC5335801

[28] Chorley BN , Campbell MR , Wang X , et al. Identification of novel NRF2‐regulated genes by ChIP‐Seq: influence on retinoid X receptor alpha. Nucleic Acids Res. 2012;40(15):7416‐7429. 22581777 10.1093/nar/gks409 PMC3424561

[29] Shah NM , Zaitseva L , Bowles KM , et al. NRF2‐drivenmiR‐125B1 and miR‐29B1 transcriptional regulation controls a novel anti‐apoptotic miRNA regulatory network for AML survival. Cell Death Differ. 2015;22(4):654‐664. 25323587 10.1038/cdd.2014.152 PMC4356334

[30] Yang D , Yuan Q , Balakrishnan A , et al. MicroRNA‐125b‐5p mimic inhibits acute liver failure. Nat Commun. 2016;7:11916. 27336362 10.1038/ncomms11916 PMC4931005

[31] Dembic Z . Cytokines of the immune system: interleukins. The Role of the Immune System.2015;143‐239.

[32] Cayabyab RG , Jones CA , Kwong KY , et al. Interleukin‐1beta in the bronchoalveolar lavage fluid of premature neonate: a marker for maternal chorioamnionitis and predictor of adverse neonatal outcome. J Matern Fetal Neonatal Med. 2003;14(3):205‐211. 14694976 10.1080/jmf.14.3.205.211

[33] Petri S , Körner S , Kiaei M . Nrf2/ARE signaling pathway: key mediator in oxidative stress and potential therapeutic target in ALS. Neurol Res Int. 2012;2012:878030. 23050144 10.1155/2012/878030 PMC3461296

[34] Joo MS , Lee CG , Koo JH , et al. miR‐125b transcriptionally increased by Nrf2 inhibits AhR repressor, which protects kidney from cisplatin‐induced injury. Cell Death Dis. 2013;4:e899. 24176857 10.1038/cddis.2013.427 PMC3920955

